# Mixed Bacterial Growth in Prenatal Urine Cultures; An Investigation into Prevalence, Contributory Factors and the Impact of education-based Interventions

**DOI:** 10.1007/s10995-023-03615-6

**Published:** 2023-03-13

**Authors:** Lindsay M Kindinger, Hannah Linton, Catherine P James, Camille Mallet, Carmel Curtis, Bruce Macrae, Anna L. David

**Affiliations:** 1 Elizabeth Garrett Anderson, Institute for Women’s Health, Faculty of Population Health Sciences, Medical School Building, University College London, 74 Huntley Street, London, WC1E 6AU UK; 2 Maternal Fetal Medicine Department, Obstetrics and Gynaecology, King Edward Memorial Hospital, Subiaco, Perth, WA, 6008 Australia; 3 Children and Young People’s General Services, University College London Hospital NHS Foundation Trust, 235 Euston Road, London, NW1 2BU UK; 4 Women’s Health Division, Elizabeth Garrett Anderson Wing, University College London Hospital NHS Foundation Trust, 25 Grafton Way, London, WC1E 6DB UK; 5 Clinical Microbiology, 5th Floor Central, University College London Hospital NHS Foundation Trust, 250 Euston Road, London, NW1 2PG UK

**Keywords:** Urinary Tract Infection, Pregnancy, Asymptomatic Bacteriuria, Mixed growth, Urine Culture

## Abstract

**Purpose:**

Undiagnosed urinary tract infections (UTIs) in pregnancy are associated with adverse perinatal outcome. Urine microbiology cultures reported as ‘mixed bacterial growth’ (MBG) frequently present a diagnostic dilemma for healthcare providers. We investigated external factors contributing to elevated rates of (MBG) within a large tertiary maternity centre in London, UK, and assessed the efficacy of health service interventions to mitigate these.

**Description:**

This prospective, observational study of asymptomatic pregnant women attending their first prenatal clinic appointment aimed to establish (i) the prevalence of MBG in routine prenatal urine microbiology cultures, (ii) the association between urine cultures and the duration to laboratory processing and (iii) ways in which MBG may be reduced in pregnancy. Specifically we assessed the impact of patient-clinician interaction and that of an education package on optimal urine sampling technique.

**Assessment:**

Among 212 women observed over 6 weeks, the negative, positive and MBG urine culture rates were 66%, 10% and 2% respectively. Shorter duration from urine sample collection to laboratory arrival correlated with higher rates of negative cultures. Urine samples arriving in the laboratory within 3 hours of collection were most likely to be reported as culture negative (74%), and were least likely to be reported as MBG (21%) or culture positive (6%), compared to samples arriving > 6 hours (71%, 14% and 14% respectively; P < 0.001). A midwifery education package effectively reduced rates of MBG (37% pre-intervention vs 19% post-intervention, RR 0.70, 95% CI 0.55 to 0.89). Women who did not receive verbal instructions prior to providing their sample had 5-fold higher rates of MBG (P < 0.001).

**Conclusion:**

As many as 24% of prenatal urine screening cultures are reported as MBG. Patient-midwife interaction before urine sample collection and rapid transfer of urine samples to the laboratory within 3 hours reduces the rate of MBG in prenatal urine cultures. Reinforcing this message through education may improve accuracy of test results.

## Significance

*What is already known on this subject?* Undiagnosed urinary tract infections (UTIs) in pregnancy are associated with adverse perinatal outcome due to elevated rates of preterm birth, maternal sepsis and low birthweight. The prevalence of mixed bacterial growth in prenatal urine cultures is poorly reported, and is of uncertain clinical significance. Mixed bacterial growth may reflect either an undiagnosed urinary tract infection (UTI) or contamination by organisms colonising the peri-urethra.

*What this study adds?* As many as one-quarter of prenatal urine cultures in a healthy pregnant population are reported as mixed bacterial growth. This inevitably adds to physician workload and patient anxiety. Key policies to reduce rates of mixed bacterial growth include ensuring urine samples reach microbiology laboratories in a timely manner (< 3 h), provision of clear verbal instructions to women on optimal urine sampling technique, and regular simple education packages for clinicians to reinforce this message.

## Background

Physiological and anatomical changes during pregnancy predispose the urinary tract to increased bacterial growth (Dafnis & Sabatini, 1992; Patterson & Andriole, 1987). The diagnostic standard for confirming clinically significant infection of the urinary tract in both pregnant and non-pregnant populations has remained unchanged for more than 70 years; the identification of bacteria at a quantity of ≥ 10^5^ colony forming units (CFU) per millilitre in urine (Kass, 1957; PHE, 2019b).

Pregnant women with urinary tract infection (UTI) may experience symptoms including frequency, urgency and micturition pain. Alternatively, symptoms may be absent even when urinary pathogens are present above the diagnostic threshold. Such patients may be classified as having asymptomatic bacteriuria (ASB) (Cormican et al., 2011; Glaser & Schaeffer, 2015). ASB has a prevalence of between 2 and 10% amongst the general pre-menopausal population (Glaser & Schaeffer, 2015) and is generally considered benign phenomenon for which treatment is not indicated (Cormican et al., 2011). In pregnancy the urinary tract is particularly vulnerable to urinary stasis and bacterial ascent to the upper urinary tract. Compared to non-pregnant populations, undetected ASB is associated with an increased risk of adverse pregnancy outcome (Farkash et al., 2012; Ipe, Sundac, Benjamin, Moore, & Ulett, 2013). Antibiotic treatment of ASB reduces the risk of pyelonephritis (RR 0.24 [95% CI 0.13 to 0.42]), rates of preterm birth (RR 0.27 [95% CI: 0.11–0.62]), low-birth-weight neonates (RR 0.64 [95% CI: 0.45–0.93]) and persistent bacteriuria in pregnancy (RR 0.30 [95% CI: 0.18–0.53]) (Widmer et al., 2015; Wingert et al., 2019). The World Health Organisation (WHO) therefore recommends a seven-day antibiotic regimen for all pregnant women with identified UTI and ASB to prevent these recognised complications (WHO, 2016).

The accurate detection of bacteriuria through screening relies on a very precise investigative process. In the UK, the National Institute of Clinical Excellence (NICE) recommends that ASB is screened for in the first trimester of pregnancy by mid-stream urine (MSU) sample collection for microbiological microscopy, culture and sensitivity (MC&S) analysis (NICE, 2008). Samples are first examined by microscopy and a definitive diagnosis is made by quantitative culture according to standards set by Public Health England (PHE) (PHE, 2019b). A positive culture reflects identification of a specific pathogen responsible for a UTI and antibiotic treatment should be dictated accordingly. *Escherichia coli* is the most common pathogen, identified in up to 80% of bacterial isolates; other pathogens include Klebsiella species, *Proteus mirabilis* and group B streptococcus (GBS) (Smaill & Vazquez, 2015). A ‘negative culture’ or ‘no significant growth’ indicates the diagnostic threshold has not been met and precludes the need for antibiotics.

A diagnostic dilemma arises when a urine sample exhibits a number of different pathogens; a phenomenon, usually described as a ‘mixed bacterial growth’ (MBG) urine culture, which is considered with the growth of up to three organisms > 10^5^ CFU/ml (PHE, 2019b). The significance of mixed bacterial growth is uncertain. It may reflect either an undiagnosed UTI with multiple pathogens or contamination by organisms colonising peri-urethral, vaginal, and perianal regions (Kass, 1957). Common practice therefore is to repeat the process of urine collection and culture when MBG has been detected (PHE, 2019a). This commits time and resources by the pregnant patient and the clinician, additional financial costs and risks potentially inappropriate antibiotic prescribing.

 The prevalence MBG in prenatal urine cultures is uncertain. Specifically, the impact of geographical location and demographic factors such as ethnicity and BMI on MBG in pregnancy is poorly reported (Bekeris et al., 2008). Studies in non-pregnant populations have demonstrated the importance of sample collection, handling, and processing on rates of MGB. Lysis of urinary leucocytes, a surrogate marker for pyuria, has been shown to occur rapidly after sample collection in non-pregnant populations thereby adversely influencing the quality of the urine study (Kupelian et al., 2013). We therefore hypothesised that the quality of prenatal urine samples and rates of MBG in pregnancy correlated with time to processing and sample collection technique. In a prospective observational study of asymptomatic pregnant women attending midwifery-led prenatal booking appointments, we investigated the prevalence of MBG, the association between sample processing duration and subsequent microbiology reports (positive, negative and MBG) and explored ways in which these may be reduced in order to improve the accurate diagnosis of ASB in pregnancy.

## Methods

This prospective study investigated the prevalence of MBG amongst urine culture results from samples provided by women attending their first prenatal booking appointment at University College London Hospital (UCLH). We also studied the impact of two variables on urine culture results: (i) the duration of time from collection of the urine sample to arrival in the microbiology laboratory, and (ii) the impact of a midwifery education package on the importance of providing women with verbal instruction on urine sampling technique in the clinic setting before providing a sample.

Throughout the study, urine collection instructions were displayed as posters in clinic bathrooms. These provided both written and visual instructions on the optimal method for women to provide a MSU sample. Data was prospectively collected over a 6 week study period (January to March 2018) from healthy pregnant women attending their first midwife-led prenatal booking appointment. Women were asked to provide a MSU sample by clinic staff as routine clinical practice. Women were provided with clean, single use specimen pots without boric acid. The time of MSU collection was recorded by an independent observer (HL) situated in the prenatal clinic, and it was noted whether urine sample collection was before or after the midwife appointment. The midwife subsequently requested microscopy, culture and sensitivity (MC&S) analysis on the sample, and placed the MSU sample in the ‘sample collection box’. The sample would sit at room temperature until hospital porters physically collected the sample at various intervals throughout the day. The time of sample receipt at the microbiology laboratory was obtained from the local pathology reporting system allowing the precise time elapsed between MSU collection and laboratory receipt to be calculated. The time of the day of the clinic and the day of the week was also noted.

Midway through the study period, the prenatal clinic midwifery staff attended an education session. This described the importance of women providing a fresh urine sample and how to instruct women on optimal midstream collection technique. Following this education intervention, observation of urine sampling continued and the time taken for MSU samples to be received in the microbiology laboratory was again calculated. MSU culture results were compared before and after the midwife education session. As per local laboratory protocol, MSU culture results were reported as negative (‘negative’ or ‘no significant growth’), positive (predominant growth of an identified organism at a quantity of ≥ 10^5^ CFU/ml or pure growth of an organism to ≥ 10^3^ CFU/ml) or MBG (significant growth of ≥ 3 identified organisms at a quantity of ≥ 10^5^ CFU/ml).

Data were analysed for correlations between MSU culture results and the time taken for MSU collection to laboratory receipt. ANOVA, Dunn’s multiple comparison and Chi squared-tests were used to compare differences in duration of MSU processing times, the impact of patient-midwife contact, and midwifery education on reported rates of ‘positive’, ‘MBG’ and ‘negative’ bacterial culture reports.

## Results

MSU culture results were recorded from 212 pregnant women attending their first prenatal booking appointment. Demographic data including ethnicity are presented in Table 1. The median maternal age was 34 years (range 19–42), BMI was 23 (range 17-45.6) and most women were primiparous (57.1%). Almost two-thirds of MSU cultures were negative 65.6% (n = 139). Bacterial isolates above the diagnostic threshold, considered as positive cultures, were identified in 9.9% (n = 21) of samples. Mixed bacterial growth of uncertain clinical significance (MBG) was reported in 24.5% (n = 52) samples (Table 1). Of these 94% (49/52) provided a further urine sample for repeat culture within 3 weeks of the original sample. No significant differences in ethnicity, maternal age, BMI or parity according to MSU culture results were observed (P > 0.05; Table 1).

**Table 1:** Demographics according to reported urine culture results

				Urine bacterial culture results					
		Total population		Negative growth		Positive growth		Mixed growth	
		N = 212		N = 139 (66%)		N = 21 (10%)		N = 52 (24%)	
Age (years)	Mean ± SD	33.1	± 4.7	33.1	± 4.7	33.6	± 4.0	32.8	± 4.8
BMI	Mean ± SD	24.0	± 4.6	25.3	± 3.7	26.6	± 6.2	25.3	± 5.2
Ethnicity, n, %	Caucasian	154	72.6%	102	66.2%	15	9.7%	37	24.0%
	South Asian	22	10.4%	14	63.6%	3	13.6%	5	22.7%
	Black	20	9.4%	12	60.0%	2	10.0%	6	30.0%
	Far East Asian	8	3.8%	6	75.0%	0	0.0%	2	25.0%
	Mixed/Other	6	2.8%	4	66.7%	1	16.7%	1	16.7%
	Unknown	2	0.9%	1	50.0%	0	0.0%	1	50.0%
Parity, n, %	Para 0	121	57.1%	79	56.8%	11	52.4%	31	59.6%
	Para ≥ 1	91	42.9%	60	43.2%	10	47.6%	21	40.4%

### Duration from MSU Collection to Laboratory Receipt

**Fig. 1:**
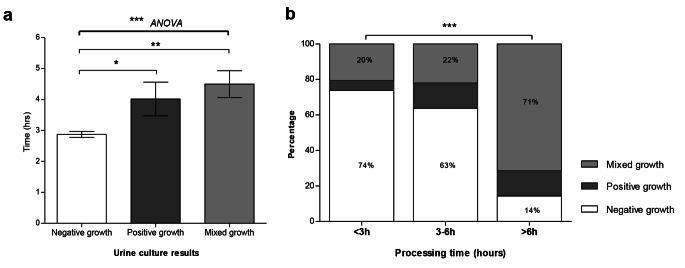
a) MSU culture result and time from urine collection to laboratory receipt (P = 0.0004; ANOVA). A mixed growth result was associated with the longest duration. Negative growth result was associated with the shortest duration b) Significantly higher rates of mixed bacterial growth (71%) and fewer negative cultures (14%) were observed among samples taking longer than 6 h to reach the laboratory. This is compared to 20% and 74% respectively of samples processed within 3 h (P < 0.0001)

The time taken from urine collection to laboratory receipt differed significantly among negative, positive and MBG urine culture reports (Table 2; Fig. 1A). Negative MSU cultures were associated with the shortest time interval (mean 2h54min [95% CI 2h42m − 3h6m]), while positive growth and MBG were associated with significantly longer durations (mean 4h0min, [95% CI 2h48m − 5h6m]) and 4h30min [95% CI 3h36m − 5h18m] respectively; P < 0.01 Dunns Multiple comparison; Table 2; Fig. 1A)

**Table 2:** Impact of time to receipt in lab, midwifery appointment and midwifery education package on reported urine culture results

	MSU culture results						
	Negative growth		Positive growth		Mixed growth		*P value*
	N = 139 (66%)		N = 21 (10%)		N = 52 (24%)		
**Time duration from MSU collection to laboratory receipt** (hours, minutes)							
Mean ± SD	2 h 54 min	± 1 h 6 min	4 h 0 min	± 2 h 30 min	4 h 30 min	± 3 h 6 min	*P = 0.0004*
Median	2 h 48 min		3 h 48 min		3 h 30 min		
Range	0 h 48 min − 7 h 24 min		1 h 12 min − 11 h 48 min		1 h 24 min − 13 h 54 min		
***Categorised time duration from MSU collection to laboratory receipt***							
< 3 h, n, %	79	73.8%	6	5.6%	22	20.6%	*P < 0.0001*
3 to < 6 h, n, %	58	63.7%	13	14.3%	20	22.0%	
≥ 6 h, n, %	2	14.3%	2	14.3%	10	71.4%	
***MSU collection in relation to midwife appointment***							
Before, n, %	27	38.0%	10	14.1%	34	47.9%	*P < 0.0001*
After, n, %	74	80.4%	9	9.8%	9	9.8%	
Unknown, n, %	38	77.4%	1	4.0%	9	18.4%	
***MSU collection in relation to education intervention***							
Pre-intervention, n, %	32	47%	11	16%	25	37%	*P = 0.005*
Post-intervention, n, %	107	74%	10	7%	27	19%	

*MSU = midstream urine; SD = standard deviation; h = time in hours; min = time in minutes*

Around half of the MSU samples arrived at the laboratory within 3 h of collection (< 3 h, n = 107/212, 50.4%). The remainder took 3 to 6 h (n = 91/212, 42.9%), or ≥ 6 h (n = 14/212, 6.6%). MSU samples arriving in the laboratory within 3 h of collection were most likely to be reported as culture negative (73.8%, 79/107), and were least likely to be reported as MBG (20.6%, 22/107) or culture positive (5.6%, 6/107), compared to samples arriving > 6 h (71.4%, 14.3% and 14.3% respectively; P < 0.001; Table 2; Fig. 1B). Receipt of the MSU sample in the laboratory ≥ 6 h was associated with a three-fold increase in MBG (RR3.5 95% CI 2.1–5.7). Despite only accounting for 6.6% of samples overall, those MSU samples that were received in the laboratory ≥ 6 h after collection accounted for 19.2% of all MBG reports.

### Timing of MSU Sample Collection

Culture reports were analysed according to whether the urine sample was provided before (n = 71) or after women had their midwife prenatal consultation (n = 92). The mean time taken to reach the lab was longer among samples provided before the midwife appointment (mean 4h24min, range 1h21min to 13h50min) compared to after the midwife appointment (mean 2h36min, range 0h51min to 7h21min; P < 0.0001).

**Fig. 2:**
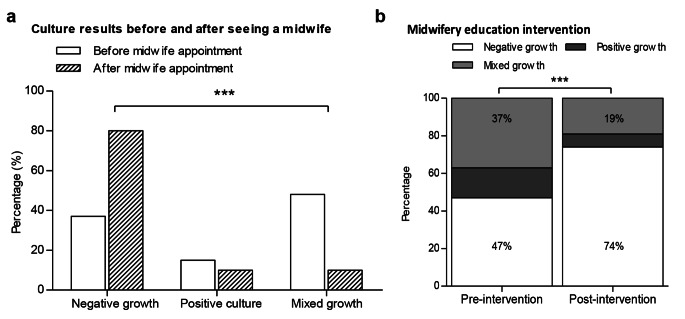
A comparison of MSU culture reports collected (a) before or after the midwife appointment and (b) pre- and post- a midwife ‘education intervention’. (a) Samples provided prior to the midwife appointment had higher rates of mixed growth (48% vs. 10%) and fewer negative cultures (37% vs. 80%) compared to those provided after the midwife appointment (P < 0.001). (b) The midwife education intervention was associated with a reduction in mixed growth (37% vs. 19%) and an increase in negative culture reports (47% vs. 74%; P = 0.0005)

Urine samples provided before the midwife appointment, and therefore before receiving any verbal instruction on sampling technique, were associated with an almost five-fold higher rate of MBG when compared to samples provided by women who had already seen a midwife (47.9% before midwife appointment vs. 9.8% after midwife appointment; RR 4.9 [95% CI 2.51 to 9.53], P < 0.001; Table 2; Fig. 2A). A corresponding three-fold increase in negative cultures were observed in samples collected following the midwife appointment compared to those collected before the appointment (80.5% vs. 38.0% respectively; RR 3.2 [95% CI 2.0 to 5.1]; Table 2; Fig. 2A).

MSU culture reports did not differ by time of day of collection. Samples taken in morning prenatal clinics (n = 37) were similar to those in the afternoon clinics (n = 175); negative cultures were reported in 70.2% of samples from morning clinics compared to 65.1% in the afternoon, positive cultures in 5.4% vs. 10.2%, and MBG in 24.3% and 25.1% respectively (P = 0.7).

### Education Intervention

An educational intervention for the midwifery staff was used to reinforce the importance of advising women at their booking prenatal appointment of the optimal MSU sampling technique, and that a fresh MSU sample provided the best culture results. This intervention occurred after data were collected from 68 women (32%) with a further 144 women (68%) studied after the education intervention. Compared to before the intervention, a decrease in MBG (37% versus 19%), a decrease in positive cultures (16% versus 7%) and a respective increase in negative cultures were observed (47% versus 74%; P = 0.0005; Table 2; Fig. 2B).

## Discussion

In our central-London cohort, approximately one-quarter of our healthy pregnant population had mixed bacterial growth reported in their routine prenatal urine sample. Although expected estimates of acceptable rates of urinary MBG in pregnant populations are poorly described, our reported prevalence exceeds averages in non-pregnant cohorts (Bekeris et al., 2008). The major factors associated with elevated rates of urinary MBG in our study were delays in transport of urine samples to the laboratory by six hours or more, as well as women providing an MSU sample prior to their face to face consultation in prenatal clinic. These factors were associated with three- and five-fold increases in reports of MBG respectively. We demonstrated that a simple education intervention for clinicians in prenatal clinic to reinforce the importance of optimal MSU collection technique substantially reduced the rates of MBG and was associated with a corresponding doubling of negative urine culture results.

Public Health England (PHE) guidance states that urine specimens should be collected hygienically and transported and processed within 4 hours (PHE, 2019b). Long delays in urine processing permit post-collection bacterial multiplication and colony formation and as a result, the urine culture may show a false positive for MBG when it is eventually processed (PHE, 2019b). Within the hospital setting obstacles often exist which make the target of less than 4 h to processing difficult to achieve. Where this is not possible, refrigeration of samples at 4 degrees Celsius can help to maintain sample integrity for up to 48 h (PHE, 2019b) and reduces rates of contamination by around 50% (Bekeris et al., 2008). Alternately where refrigeration is not possible, bacterial colony preservation may be achieved through the addition of boric acid. Boric acid converts urine into an effective bacteriostatic transport medium, preventing degradation of white cells, overgrowth of organisms (Meers & Chow, 1990), and appears to be superior to refrigeration for the preservation of urine quality for up to 25 h post collection (Kupelian et al., 2013). Urine sample tubes with boric acid are commercially available, but the associated high cost, and inability to perform a dipstick may prohibit widespread use in the prenatal setting. Boric acid inevitably adds to the collection workflow, as the urine sample needs to be decanted into the boric acid tube following a positive urine dipstick, thereby introducing additional space and time requirements for the healthcare practitioner.

NICE recommends that all women in prenatal clinic settings receive instruction on the optimal midstream urine collection technique (NICE, 2008) to reduce contamination rates (Bekeris et al., 2008). Our study provides evidence that this guidance should be reinforced through regular staff education sessions. We found that an education intervention for midwifery staff on the importance of providing verbal instructions on optimal MSU collection to pregnant women was associated with a five-fold reduction in reports of MBG and a corresponding two-fold increase in negative cultures. A systematic review of further pre-analytic practices found that perineal cleansing may also reduce MSU culture contamination, although pregnant women were not included as a specific group in this review (LaRocco et al., 2016). Other older studies in prenatal cohorts indicate this is not likely to be effective (Holliday et al., 1991; Schlager, Smith, & Donowitz, 1995).

More sophisticated techniques including sediment culture and 16SrDNA PCR techniques have been shown to be superior to standard culture (Sathiananthamoorthy et al., 2019). These may be used in future to better evaluate bacteriuria, but is unlikely to overcome the challenge posed by mixed growth secondary to contamination. The use of a MALDI-TOF MS (Matrix-Assisted Laser Desorption/Ionization Time of Flight Mass Spectrometry) to rapidly assess bacterial isolates and rank them by pathogenicity provides improved identification of causative bacteria in complicated UTIs (Kitagawa et al., 2018). This tool has been shown to provide rapid and accurate diagnoses for routine UTIs and may offer a feasible substitute for conventional urine cultures in the future (Kitagawa et al., 2018).

In the absence of these techniques it remains common practice for clinicians to repeat MSU cultures following a MBG report, in an attempt to differentiate between contamination of a negative culture and an undiagnosed ASB. A proportion of patients with true (or clinically significant) bacteriuria may be left untreated in the interim. This exposes them to an associated risk of subsequent acute pyelonephritis and preterm birth (Wingert et al., 2019). An alternative option to negate the risks of undetected ASB, is to treat the MBG immediately, albeit risking inappropriate antimicrobial use in the absence of infection. Already it is estimated that up to 40% of women receive antibiotics during pregnancy (Lamont et al., 2014) with increasing concerns about antibiotic resistance (Rizvi et al., 2011).

### Strengths and Weaknesses

The strength of our study is that we prospectively observed routine practice and through a simple education program, that is easy to repeat, we demonstrated an impact on a common and costly routine booking test in pregnancy. We were able to achieve complete outcome data for the times of laboratory processing through having an observer in the prenatal clinic. We did not investigate the effect of boric acid tubes or refrigeration facilities in the prenatal clinic as we were most interested in a behavioural intervention which is very low cost to implement.

The main limitation of this study is its uncertain generalisability. Little is known about the prevalence of urinary MBG in pregnancy, whether our findings are representative of wider populations and if our interventions are reproducible in other cohorts. Additionally, insufficient evidence exists as to conclude whether mixed growth constitutes a clinically significant prenatal finding (Naresh & Simhan, 2011). Our study did not set out to investigate the clinical impact of the education package on pregnancy outcomes such as pyelonephritis, sepsis, preterm birth or neonatal outcome. Further studies are warranted to provide a broader understanding of the impact of MBG in pregnancy.

## Conclusion

We demonstrate a high prevalence of mixed bacterial growth within prenatal urine cultures. Healthcare providers may reduce the burden of mixed growth urine culture results at prenatal appointments through ensuring samples reach the microbiology laboratory in a timely manner and through provision of clear verbal instructions on optimal sampling technique before women provide their urine sample. A simple education package for healthcare providers is effective at reinforcing this message. There may be an impetus for introducing refrigeration of urine samples or preservation with boric acid at the point of collection to reduce the amount of post-collection bacterial growth.

## Data Availability

Not applicable

## List of abbreviations

CFU: Colony Forming Units
UTI: Urinary Tract Infection
ASB: Asymptomatic Bacteriuria
MSU: Mid-stream Urine
MBG: Mixed bacterial growth
MC&S: Microscopy, Culture and Sensitivity
NICE: National Institute of Clinical Excellence
PHE: Public Health England
UCLH: University College London
